# Metformin Inhibits Mitochondrial Complex I To Promote Health

**DOI:** 10.1093/geroni/igab046.1760

**Published:** 2021-12-17

**Authors:** Navdeep Chandel

**Affiliations:** Northwestern University, Illinois, United States

## Abstract

The major function of mitochondria in cellular homeostasis has been the generation of ATP through oxidative phosphorylation. However, we have previously demonstrated that mitochondria can serve as signaling organelles by releasing low levels of reactive oxygen species (ROS) and TCA cycle metabolites that are essential for hypoxic activation of HIF, antigen activation of T cells, cellular differentiation and proliferation of cancer cells. The anti-diabetic drug metformin has been proposed to inhibit mitochondrial complex I. We will present data indicating that metformin inhibits mitochondrial complex I to exert it’s biological effects through controlling ROS, ATP, and NAD+.

